# Traditional Uses of Leguminosae among the Karen in Thailand

**DOI:** 10.3390/plants8120600

**Published:** 2019-12-13

**Authors:** Natcha Sutjaritjai, Prasit Wangpakapattanawong, Henrik Balslev, Angkhana Inta

**Affiliations:** 1 Department of Biology, Faculty of Science, Chiang Mai University, Chiang Mai 50200, Thailand; natcha.sutjaritjai@gmail.com (N.S.); prasitwang@yahoo.com (P.W.); 2 Department of Biological Sciences, Aarhus University, DK-8000 Aarhus, Denmark; henrik.balslev@bios.au.dk; 3 Center of Excellence in Bioresources for Agriculture, Industry and Medicine, Chiang Mai University, Chiang Mai 50200, Thailand

**Keywords:** cultural importance index, ethnic groups, ethnobotany, fabaceae, fidelity level, legumes, local scale, plant usage, Skaw Karen, traditional knowledge

## Abstract

Leguminosae (legumes) are one of the largest plant families. They are widely used for a variety of purposes by people around the world and include many important cultivated economic food crops. On local scales, legumes are commonly used by various ethnic groups. However, the data are incomplete and scattered, not least in Thailand. We found that species of legumes were important in Karen communities, so we decided to investigate in detail the traditional knowledge of legumes on a local scale among Karen people in northern Thailand. We interviewed six herbalists and eighty-four nonherbalist informants in three Karen villages in Chiang Mai province about their use of legumes, and about the local names for the species, using semistructured interviews. A total of 83 legumes species (in 45 genera) had 4443 use reports. Five of the 83 legume species had not been reported previously as used in Thailand. Most Karen use reports (43%) of legumes were for food, medicine (36%), and materials (8%), but in term of species more legumes (68 species) were used for medicine than for food (53 species). The legume genera with most used species were *Crotalaria* and *Flemingia* each with six species. The most important species are *Tamarindus indica* (CI = 3.38), *Senegalia rugata* (CI = 2.39), *Glycine max* (CI = 1.27) respectively.

## 1. Introduction

Leguminosae (legumes) are the third largest plant family, with approximately 19,400 species [1], and they are found throughout the world in all biomes [2]. In Thailand, this family comprises about 133 genera and 778 species [3,4,5]. Legumes include many useful plants, such as crops, vegetables, timber, ornamentals, and medicinal plants [6], and are also important as fodder and green manure [7]. On a global level, many legumes are grown as economic crops. Legumes are the second most important crop family following the Poaceae [8].

Legumes are also important at the local scale. Many ethnobotanical studies in Thailand have demonstrated that legumes have always had more uses and used species than other plant families [9,10,11]. Unfortunately, many ethnobotanical studies in Thailand are focused only on single ethnic groups and many are unpublished. Therefore, information about legume uses in Thailand remains incomplete. Because legumes are so important at all scales, it is important to document their uses.

The Karen is the largest ethnic minority group in Thailand. They have migrated from Myanmar and settled in the northern and western parts of Thailand since the eighteenth century [12]. The largest proportion of the Thai Karen population live in northwestern Thailand in Chiang Mai, Mae Hong Son, and Tak provinces [13]. The Karen community’s livelihood depends on agriculture and livestock farming. They typically live surrounded by natural forest in the mountains. The Karen lifestyle still relies on nature, and they maintain large proportions of their traditional knowledge [14]. Therefore, the Karen are good representatives for studying local ethnobotanical uses of legumes.

Considering the global importance of legumes, in combination with the limited research into their local uses among ethnic groups, we expect many unreported local uses of legumes and that they may be underutilized. New studies that focus on legumes and on discovering their local uses may expose their overall usefulness. To do so, we focus on ethnobotanical uses of legumes in Karen communities in Thailand. Specifically, we asked the following questions: (1) How many legume species do the Karen use? (2) Which uses of legumes remain unreported in previous ethnobotanical studies? (3) Which use categories are the most important? (4) In which habitats do the useful legumes grow, which lifeform do they have, and which part of the plants are used? Finally, to rank the species we calculated the cultural importance index (CI) and to estimate the distribution of traditional knowledge in the communities we calculated the fidelity level (FL) for each legume encountered.

## 2. Results

### 2.1. Traditionally Used Leguminosae

A total of 83 legume species in 45 genera were used by the Karen and mentioned by the six key informants and the 84 nonspecialists from three studied villages (Table 1 and Table 2). The genera *Crotalaria* and *Flemingia* had the most useful species (6 in total), followed by *Senna* and *Vigna* (5 in total) (Table 2).

### 2.2. Previously Unreported Legume Uses

Comparing with previous ethnobotanical studies of the Thai Karen [15,16,17,18,19,20], we found 17 species of legumes that had not been recorded as useful among the Karen (Table 2). Some of the species that had not been recorded for the Karen, however, did have records of use among other ethnic groups. For example, *Bauhinia purpurea* L. is commonly consumed by Tai Yuan [21], and here we documented that the Karen cultivated it in their home gardens and used its young leaves as a vegetable. For five of the legume species recorded here (*Aeschynomene americana* L., *Crotalaria lejoloba* Bartl., *Flemingia paniculata* Benth., *Indigofera hendecaphylla* Jacq., and *Vigna dalzelliana* (Kuntze) Verdc.) we could not find any report of traditional use elsewhere in Thailand [22].

### 2.3. Use Categories 

**Figure 1:**
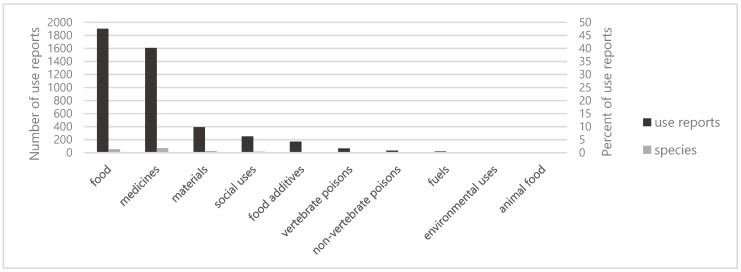
Number and percentage of use reports of Leguminosae in in each use categories in three Karen villages in northern Thailand.

Our fieldwork generated 4443 use reports for the 83 legume species mentioned by ninety informants, including six specialists and 84 nonspecialists, from the three Karen communities in Thailand (Table 2). Khuntae had the highest total number of use reports (1609, 36%), followed by Pakanok (1469, 33%) and Tuan (1365, 31%) (Table 1). The uses of the legumes belonged to ten use categories in the system of Cook (1995) [23]. Food (1901 use reports; 43%), medicine (1608 use reports; 36%), and materials (390 use reports; 8%) were the three most important use categories (Figure 1).

### 2.4. Habitats, Life Forms, and Parts Used

Over half (56 species) of the useful legumes were found in the forests surrounding the villages, whereas other habitats were less important, such as village areas (20 species), home gardens (14 species), and agricultural areas (13 species). Most of the legumes used for food were, not surprisingly, found in the agricultural fields (e.g., *Phaseolus vulgaris* L., *Vigna mungo* (L.) Hepper, and *Vigna umbellata* (Thunb.) Ohwi and H.Ohashi) and home gardens (e.g., *Cajanus cajan* (L.) Millsp., *Canavalia ensiformis* (L.) DC., and *Lablab purpureus* (L.) Sweet). Medicinal plants and plants in other use categories were found both in natural forests (e.g., *Crotalaria alata* D.Don, *Phyllodium longipes* (Craib) Schindl, and *Xylia xylocarpa* (Roxb.) Taub.) and in the village areas (e.g., *Aeschynomene americana* L., *Indigofera hendecaphylla* Jacq., and *Millettia caerulea* Baker).

**Figure 2:**
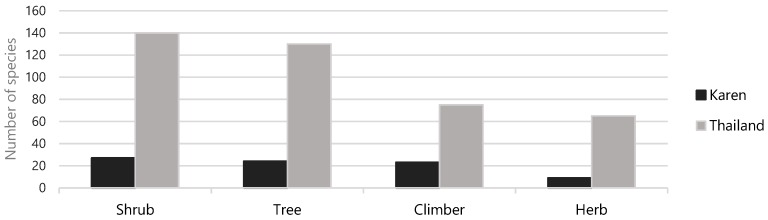
Number of species of Leguminosae in each life form with traditional uses in three Karen villages in northern Thailand compared to their total number of species in each life form in the Thai legume flora.

Legume species may comprise various life forms. Among the legumes used in the three Karen villages, shrubs were the most common life form, followed by trees, climbers, and herbs, which corresponds to the proportion of the same life forms in the Thai legume flora (Figure 2).

For the parts used, the Karen most often used the leaves of the legumes (22%). Otherwise, the proportion of used parts were fruits (20%), seeds (15%), roots (12%), stems (9%), whole plants (8%), bark (7%), inflorescences (6%), and exudates (1%). The leaves were used mostly for food and medicine. The fruits, the seeds, and the inflorescences were generally used for food. The roots, the stems, and the whole plants were often used for medicine, and the stems were popular for materials. The exudates were used for social and medicinal purposes (Table 2). 

### 2.5. Cultural Importance Index (CI) and Fidelity Level (FL) 

The cultural importance index (CI) for legume species used by the Karen varied from 0.02 for *Mimosa diplotricha* to 3.38 for *Tamarindus indica* (Table 2). The highest CI value for a legume used by the Karen was for *Tamarindus indica* (CI = 3.38), the second was for *Senegalia rugata* (CI = 2.39), and the third was for *Glycine max* (CI = 1.27). The CI value ranked how important the 83 legume species were to the Karen communities (Table S1). 

The fidelity level (FL) showed the informants’ consensus about the use of legumes in each category (Table 2). The highest value that can be obtained of the fidelity index is one hundred. Lower number of fidelity value means fewer uses in that use categories and less agreement about the uses among the informants. Obtaining a hundred percent fidelity level means that a species is used in only one category. The species that were used in only one category by the Karen, such as *Aeschynomene americana* L., *Flemingia congesta* Roxb. Ex W.T. Aiton, *Huangtcia renifolia* (L.) H.Ohashi and K.Ohashi, and *Uraria oblonga* (Wall. ex Benth.) H.Ohashi and K.Ohashi, were all used exclusively for medicine. Many of the edible species were only used for food, for example, *Bauhinia variegata* L., *Phaseolus vulgaris* L., and *Vigna umbellata* (Thunb.) Ohwi and H.Ohashi. The calculation of FL value showed in Table S2.

## 3. Discussion

### 3.1. Traditionally Used Leguminosae

Among the 45 useful legume genera, *Crotalaria*, *Flemingia*, *Senna*, and *Vigna* had the most useful species. *Crotalaria* is common in Thailand, where it is represented by 38 species [24]. At a global level, *Crotalaria* species are used for food and green manure, and are consumed by humans throughout the tropics. *Crotalaria* plants have a high nutrient content and they contain starch, protein, dietary fiber, oligosaccharides, and several active compounds and minerals [25]. *Crotalaria* is also popular for cultivation as green manure to improve soil quality. *Crotalaria juncea* L. and *C. trichotoma* Bojer are used elsewhere in this manner [26,27]. We found that all *Crotalaria* species in the Karen villages we studied were used for medicinal purposes, which was different from their popular uses at a global level. Because *Crotalaria* species contain useful secondary compounds, such as alkaloids, saponins, and flavonoids, they can be used as medicine [28]. *Flemingia* and *Senna* are commonly found in the villages, making them easy to obtain and use by villagers [29], and both genera are much used in traditional medicine. *Flemingia* mainly has flavonoids, which are useful in health care, whereas *Senna* has a variety of bioactive compounds that stimulate the digestive system, such as sennosides, glycosides, and naphthalene glycosides [30,31]. *Vigna* contains many species used as food in many local communities [32].

The village Khuntae had the highest number of use reports, possibly because it is a big community and quite isolated from urban centers. In the past, Khuntae could only be reached by a dirt road on steep slopes in the mountains. The paved road to Khuntae was finished as recently as in 2016. The isolation from urban communities forced the Khuntae villagers to maintain their traditional knowledge of useful legumes more than was necessary for the other two study villages. The Pakanok and Tuan villages had nearly the same distances to the nearest urban centers (Table 1) but the access to Pakanok is still along a dirt road. Therefore, Pakanok villagers are less affected by urban civilization than the villagers of Tuan. Other studies have found that the further away from urban centers, the more traditional knowledge is maintained [33,34].

### 3.2. Previously Unreported Legume Uses

We found 17 useful legume species that had not previously been reported among the Karen and five species that had no previous uses recorded among any other Thai ethnic group. Some species may have been overlooked in previous studies, which often gave more attention to the surrounding forest area. Several legumes were found in agricultural fields and home gardens, such as the cultivated crops *Vigna mungo* (L.) Hepper and *Vigna umbellata* (Thunb.) Ohwi and H. Ohashi. The discovery of five new useful legume species shows that a study of traditional uses focusing on one taxonomic group may help the informants to remember more uses for each species. Ethnobotanical studies that are focused on a particular plant group can, therefore, find species with previously unreported uses and which may be underutilized.

### 3.3. Use Categories 

At a global level, legumes are commonly mentioned as important food crops [7,35]. In our local scale study, the most important use category for legumes was medicine. In total, 68 medicinal legume species were recorded in this study, which was an outstanding number among the studies of medicinal uses of legumes. Studies of medicinal legumes from Argentina and Chile reported 35 species [36], from Bangladesh 32 species [37], and from India 50, 28, 20, and 14 species, respectively [38,39,40,41]. The Karen used many medicinal legumes probably because they still maintained their traditional knowledge of medicinal plants. Additionally, legumes were common in the natural forests surrounding many villages and they are known to contain many useful bioactive components [42]. In a recent study of traditional medicinal plant diversity in Thailand [11], Leguminosae was represented by more species than any other plant family. The food plants were represented by 53 species. In general, legumes are important food plants for low income people [43]. In the study villages, the Karen grew legumes in paddy cultivation areas and harvested them at the same time as the paddy rice, and kept some seeds for growing the following years. The food category has more use reports but has a lower number of species, surely because the food plants are used on a daily basis and they were mentioned by almost all the informants. Medicine legumes, in contrast, have more specific uses and were mentioned by fewer informants.

### 3.4. Habitats, Life Forms, and Parts Used 

Most of the useful legumes were found in the forests, which showed that the Karen livelihood remained highly dependent on the natural vegetation surrounding their villages, as pointed out in previous studies of this ethnic group [18]. The habitat of useful legumes is related to their uses. The legumes used for food were found mostly in the agricultural fields, because there the villagers could harvest them easily for daily consumption. Medicinal plants and plants in other use categories were found both in the natural forest and in the village areas, reflecting that they were used less often.

The legumes have various life forms. Shrubs appear in all seasons, and it is easy to collect all parts of them throughout the year. This may be the reason why shrubs were the most commonly used by the Karen. Legume trees were also present in all seasons, but their height sometimes made it hard to collect their leaves and flowers or fruits for use. Some legume climbers and herbs are dormant during the dry season and flourish again after the rain starts. This seasonal appearance may have affected the proportion of used species, especially of herbs, which are simply not available in parts of the annual cycle. Finally, the number of used species in each lifeform was influenced by the size of the species pool in each of the lifeform categories (Figure 2).

The reason why Karen people used the leaves of legumes may be because it is convenient and easy. Legume leaves can be used for many purposes, especially for food and medicine. On a global level, in many ethnobotanical studies, leaves are the most commonly used [44,45,46,47] because they are abundantly available. In comparison, in an ethnomedicinal study of Acanthaceae in Africa, including herbs, shrubs, and climbers, the most commonly used part was the leaf, while the whole plants was also commonly used [48]. For Annonaceae, most of which are trees, a study in Africa showed that the commonly used parts were bark and stem [49]. The most popular plant parts used depend on the plant habit. As legumes have various life forms, the parts used depends on the convenience of use. Fruit and seed are the most commonly used parts of legumes for consumption.

### 3.5. Cultural Importance Index (CI) and Fidelity Level (FL)

The cultural importance index (CI) ranks the species according to their value to the community [50,51]. When measured by their CI value, *Tamarindus indica* (tamarind) was the most important legume in the Karen communities. The tamarind was mentioned as useful by every informant, and it was used in six different use categories. The Karen used tamarind for food, food additives, fuel, materials, medicines, and for social uses. The tamarind was commonly found in the village areas, where it was easy to access and it had high use value [52]. *Senegalia rugata*, the second most important legume in the Karen communities, was easily found in the villages, and it was used for many purposes. such as food, food additives, materials, medicines, and also social uses, including Karen rituals where they paid respect to elderly people and evicted wickedness. *Glycine max* (soybeans), the third most important legume, was used for food and food additives by almost all informants. The Karen grow and harvest seeds for food and make traditional fermented soybeans.

The fidelity level (FL), which shows the evenness of traditional knowledge about a particular plant among the villagers, was calculated for each species to indicate the preference of Karen people. The fidelity level could be used to determine which species should be studied in more detail in the future, for instance to discover if they possess bioactive compounds.

## 4. Materials and Methods

### 4.1. Study Site

**Figure 3:**
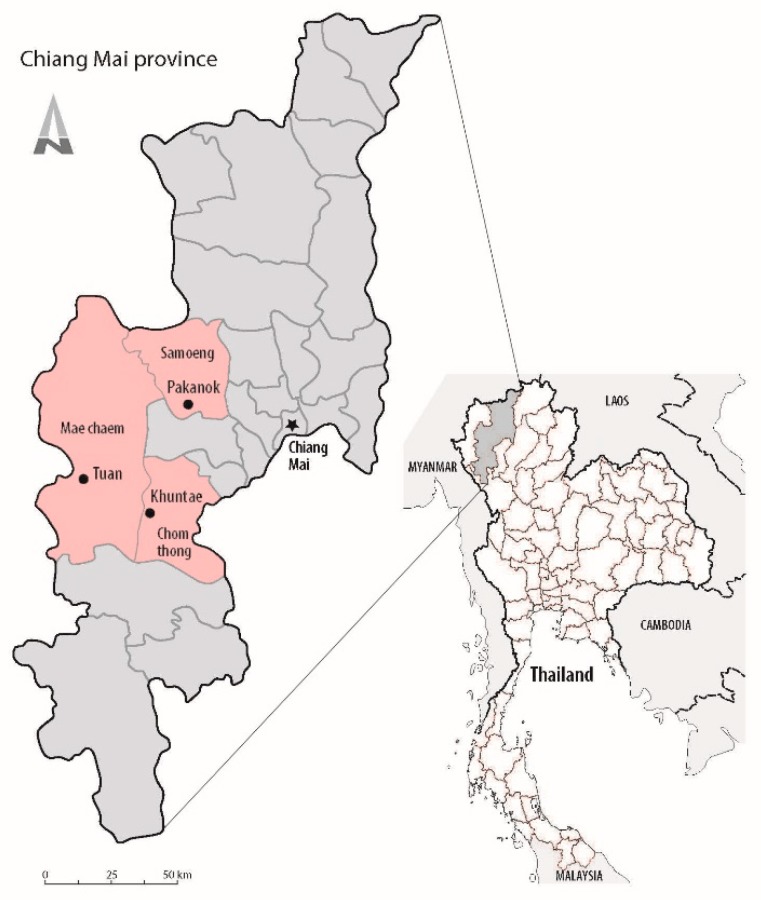
Location of three Karen villages in Chiang Mai province, Thailand, where traditional uses of Leguminosae were studied.

The field work was done in three Karen villages in Chiang Mai province, northern Thailand (Figure 3), in districts with high Karen populations: Tuan in Mae Chaem district, Pakanok in Samoeng district, and Khuntae in Chom Thong district. Their distances from the nearest urban centers range 15–26 km, and they are located at elevations ranging 863–1265 m above sea level (Table 1). All three study villages are surrounded by natural vegetation of mixed deciduous and dry evergreen forests. Most of their villagers practice agriculture, planting upland rice and some vegetables, and they raise livestock for consumption and sale. All villages have electricity and public upland water resources.

### 4.2. Data Collection

Traditional use data concerning legumes were collected from 90 informants in three Karen villages between July 2016 and December 2018. The research protocol was approved by Chiang Mai University Research Ethics Committee with the certificate of approval number certificate of analysis (COA) No. 018/61. The key informants were selected for their traditional knowledge, and because they were recommended by the village headmen and other villagers. The key informants were interviewed through field interviews and semistructured interviews [53]. Field interviews were done in the areas around the villages in forests, home gardens, agricultural areas, and village areas. The key informants walked through the areas and told us about legumes usage. The data were collected from semistructured interviews, including their local names, the plant parts used, and when the plants had medicinal uses, the mode of preparation and how the derived medicines were applied. The key informants were interviewed in Thai language or through a translator when they could not communicate in Thai. The plants were photographed, and voucher specimens were collected and deposited in the Queen Sirikit Botanic Garden Herbarium (QBG), Chiang Mai, Thailand. The plants were identified using standard taxonomic literature at the Ethnobotany and Northern Thai Flora Laboratory, Chiang Mai University.

All legume use data derived from the interviews, with the six key informants used to prepare a questionnaire for interviewing nonspecialist villagers. Twenty-eight nonspecialist villagers were selected in each village by snowball sampling methods [54]. The snowball sampling method began with interviewing the key informants and asking them to suggest the next informant who had traditional knowledge about plant use. The informant who was suggested by key informants suggested the next informant continuously until the total number of all informants reach 30 in each village. Plant pictures were shown and Karen plant names were mentioned to each of the informants. Questions were asked individually about legume uses, including their local names, the plant parts used, and routes of administration. Plant use data were categorized following the Economic Botany Data Collection Standard [23]. The plant uses recorded covered 10 categories, namely (1) food: human food, including beverages; (2) food additives: processing agents and additional ingredient that are used for seasoning during food preparation; (3) animal food: forage and fodder for vertebrate animals; (4) materials: primary staples for making derived products; (5) fuels: woods, fibers, petroleum substitutes, fuel alcohols; (6) social uses: used for social purposes; (7) vertebrate poisons: plants that are poisonous to vertebrates; (8) nonvertebrate poisons: plants that are poisonous to nonvertebrates; (9) medicines: plants that are used to treat illness both in humans and domestic animals; (10) environmental uses: plants that are used to improv the environment.

### 4.3. Data Analysis

Ethnobotanical indices were used to analyze the traditional knowledge about legumes to determine which species were the most important to the Karen communities. The cultural importance index (CI) ranks the species according to their value to the community; the value is high when a species is often mentioned by informants and when it can be used in various use categories [49,50]. The cultural importance index (CI) [55] was calculated based on data obtained from the key informants as:

CI = ∑(*u* = 1)*^NC^* ∑(*i* = 1)*^N^ UR_ui_*/N

where NC is the total number of use categories for species i, URui is the total number of use reports of each category, and N is the total number of informants. The value of CI varies between zero and the number of use categories. A species is more important when its CI value is high. The CI value was calculated with common Excel spreadsheet (Microsoft Excel for Office 365, 2014).

The fidelity level is an indication of agreement among informants about the use of a species. The highest value that can be obtained for the fidelity level is one hundred. Lower values of fidelity level mean that fewer informants knew the uses of a species in a use category. Obtaining one hundred percent fidelity level means that a species is used in only one category. The fidelity level was calculated for each species to indicate the popular use categories and to show the potential of each species. The fidelity level [56] was calculated as:

FL (%) = (Np/N) × 100

where Np is the number of use reports in each of the use categories and N is the total number of use reports. The summed fidelity level value for all plant is one hundred. High fidelity level value means that a use category is more popular. The Fidelity Level value was calculated with common Excel spreadsheet (Microsoft Excel for Office 365, 2014).

## 5. Conclusions

A total of 83 species in 45 genera of legumes had traditional uses in the three Karen villages. Five of the species had never before been documented for their traditional use in Thailand, and 17 were reported for the first time for traditional uses among the Karen. Most of the legumes were used as medicinal plants, which differed from the general appreciation of legumes as important food plants at the global level. Our study confirms that legumes are economically important, not only at the global level, but also at the local scale. Encountering so many new uses of the legumes demonstrated the value of focusing on a particular plant group in ethnobotanical studies. We suggest that the traditional uses of legumes should be studied in more details among other ethnic groups to discover additional useful species. Our future research will be focused on medicinal uses of legumes to investigate which species of legumes among the Karen communities have therapeutic potentials that can be developed further in pharmacology.

## Figures and Tables

**Table 1:** Basic information for three Karen villages in northern Thailand where traditional uses of Leguminosae were studied.

Village Name	District	Coordinates	Elevation (m a.s.l.)	Distance from Nearest Urban Center (km)	#Households	#Inhabitants	#Informants		Gender		Informant Age Range	#Use Reports	
							Specialist	Nonspecialist	F	M		Average/Informant	Total
Khuntae	Chom Thong	18 23′ 29″ N 98 30′ 23″ E	1228	26	229	807	2	28	15	15	45–80	54	1609
Pakanok	Samoeng	18 46′ 5″ N 98 39′ 35″ E	863	17	67	278	2	28	15	15	43–95	49	1469
Tuan	Mae Chaem	18 30′ 53″ N 98 16′ 33″ E	1265	15	73	253	2	28	15	15	34–83	46	1365

**Table 2:** Qualitative and quantitative data on Leguminosae species with traditional uses, registered in three Karen villages in northern Thailand.

Species [Karen Name]	CI Value	Voucher N.Sutjaritjai no.	Life Form	Habitat	Use Categories	Part Used	Method of Preparation	Application (Route of Administration Only in Medicine Category)	FL (%)
*Adenanthera*									
*Adenanthera pavonina* L. [Sa lae khwo]	0.91	0570	T	F	F	L	bo	vegetable	78
					Ma	Fr	cw	shampoo	11
					Me	Fr/L	bo	puerperium (bath)	11
*Aeschynomene*									
*Aeschynomene americana L. [Sae ya po]	0.57	0628	S	F/V	Me	W	de	back pain/lumbago (potions)	100
						W	de	muscle relaxant (potions)	
*Afzelia*									
**Afzelia xylocarpa (Kurz) Craib [Sae ki mae]	0.80	0522	T	F/M	F	Se	ro	snack	7
					Ma	Se	re	necklace beads	8
					Me	Se	gr	poisonous insect bites (liniment)	75
					SU	Se	ts	chew with betel nut	10
*Albizia*									
*Albizia chinensis* (Osbeck) Merr. [Sa per mu]	0.77	0779	T	F	Ma	B	cw	detergent/soap (cleanser)	85
					Me	B	cw	antidandruff (wash hair)	15
*Albizia procera* (Roxb.) Benth. [Pu wu / Kwa]	0.32	0630	T	F	F	L	bo	vegetable	48
					Fu	St	cu	firewood	3
					Ma	St	cu	musical instruments	14
					Me	B/R	de	coughs (potions)	35
*Arachis*									
*Arachis hypogaea* L. [Poe toe or koe]	1.00	0979	H	A	F	Se	bo/co	snack/dessert	100
*Archidendron*									
*Archidendron clypearia* (Jack) I.C.Nielsen [Cho tu mae]	0.66	0780	T	F	Fu	St	cu	firewood	3
					Ma	L	bm	gun powder	22
					Me	L	bm	blisters and burns (poultices)	70
					NP	L	no	insect repellents	5
*Archidendron jiringa* (Jack) I.C.Nielsen [Sa nae sa]	0.63	0706	T	H/M	F	Se	pr/bo	vegetable	90
					Me	B	de	flatulence/food poisoning (potions)	10
*Bauhinia*									
**Bauhinia purpurea L. [Ger her kwo]	0.23	0220	T	H	F	Fl/L	co	vegetable	100
*Bauhinia variegata* L. [Ger her por]	0.99	0981	T	F	F	Fl	co	vegetable	100
*Biancaea*									
***Biancaea sappan (L.) Tod. [Khwo]	1.07	0601	T	F	F	Fr	co	vegetable	1
					Ma	St	cu	construction materials	1
					Me	B/St	de	potionsanemia/puerperium	98
*Cajanus*									
***Cajanus cajan (L.) Millsp. [Glae bue sa]	1.07	1015	S	H/F	F	Fr/Se	bo	snack	87
					Me	L	cr	scabies (liniment)	13
**Cajanus goensis Dalzell [Mi yor por]	0.49	0761	C	F/V	F	Fl/Fr	no	snack	100
*Canavalia*									
**^,^***Canavalia ensiformis (L.) DC. [Bo ba ji noe]	0.76	0697	C	H	F	Se	co	vegetable	100
***Canavalia gladiata (Jacq.) DC. [Bo ba per yor]	0.18	1186	C	H	F	Fr/Se	co	vegetable	100
*Cassia*									
*Cassia fistula* L. [Poe yow]	0.78	0565	T	H/V	F	L	bo	vegetable	10
					Ma	B	cw	soap	1
					Me	Fr/Se	de	purgative (potions)	89
*Crotalaria*									
**Crotalaria alata D.Don [Jae gwae po]	0.18	0819	S	F	F	Se	ro	snack	6
					Ma	St	dr	broom	12
					Me	R	de	urethral stones (potions)	63
					SU	Fl	no	worship flower for ritual	19
**Crotalaria albida Roth [Jae gwae po]	0.04	0633	S	F	Me	W	de	back pain (potions)	100
						W	de	lumbago (potions)	
**Crotalaria bracteata DC. [Jae gwae po]	0.23	0677	H	F/V	Me	W	de	back pain/lumbago (potions)	86
					SU	Fl	no	worship flower for ritual	14
*Crotalaria lejoloba Bartl. [Jae gwae po]	0.19	0665	H	F/V	F	Fr	no	vegetable	6
					Me	R/W	de	back pain/lumbago (potions)	76
					SU	Fl	no	worship flower for ritual	18
**^,^***Crotalaria pallida Aiton [Jae gwae po]	0.11	0760	H	F	F	W	bo	vegetable	10
					Me	R	de	urethral stones (potions)	80
					SU	Fl	no	worship flower for ritual	10
**Crotalaria sessiliflora L. [Jae gwae po]	0.04	0634	H	F	Me	W	de	back pain (potions)	100
						W	de	lumbago (potions)	
*Dalbergia*									
*Dalbergia cultrata* Benth. [Sae klui]	0.76	0659	T	F/V	AF	L	no	cattle fodder	1
					F	L	bo/co	vegetable	15
					Fu	St	cu	firewood	2
					Ma	St	cu	construction materials	54
					Me	St	de	diarrhea (potions)	28
*Dalbergia ovata* Benth. [To gloe boe]	0.62	0766	T	F	Me	B	no	aphthous ulcer (chew/lozenge)	75
					SU	B	no	chew with betel nut	25
*Dalbergia stipulacea* Roxb. [Se ja]	0.16	0781	S	F	Fu	St	cu	firewood	7
					Ma	St	cu	fence	50
					Me	B	de	muscle relaxant/lumbago (potions)	43
*Desmodium*									
*Desmodium velutinum* (Willd.) DC. [Nor jor bi]	0.72	0695	S	F	Me	R/W	de	back pain/lumbago (potions)	98
					NP	L	no	insect repellents	2
*Dunbaria*									
*Dunbaria bella* Prain [Sae tor nor eu]	0.38	0940	C	F	F	Fl	bo/no	vegetable	88
					Me	L	de	flatulence (potions)	12
*Entada*									
*Entada rheedii* Spreng. [Mi ri gae]	1.19	0774	C	F/V	F	Fl/L	bo	vegetable	41
					Ma	B	cw	shampoo	31
					Me	Se	bm	cataracts in human or cattles (blow)	26
					SU	Se	no	pray for ritual and believe	2
*Eriosema*									
*Eriosema chinense* Vogel [Tii si go po]	0.69	1175	H	F	F	R	no	fruit	57
					Me	R	no	diarrhea (oral)	40
					SU	R	no	chew with betel nut	3
*Erythrina*									
*Erythrina stricta* Roxb. [Choe co]	0.31	1037	T	F	F	Fl/L	bo	vegetable	50
					Ma	St	cu	bucket	46
					Me	Fl	co	stomachache (oral)	4
*Erythrina subumbrans* (Hassk.) Merr. [Choe tee]	0.43	1073	T	F	EU	W	no	prevent soil erosion	8
					F	Fl/L	bo	vegetable	56
					Ma	St	cu	bucket	23
					Me	R	cu	anti-alcohol poisoning (put in the mouth)	13
*Flemingia*									
*Flemingia congesta* Roxb. Ex W.T. Aiton [Chor ae go boe]	0.58	0958	S	F	Me	L	po	wounds (poultices)	100
						R/W	bo/de	fatigue/jaundice (bath/potions)	
*Flemingia lineata* (L.) Aiton [Chor ae go boe]	0.18	1135	S	F	Me	R	bo/de	typhoid (bath/potions)	100
						W	bo/de	puerperium (bath/potions)	
*Flemingia paniculata Benth. [Chor may hmo boe]	0.48	0650	S	F/V	Me	L/R	de	appetite stimulant (potions)	100
						W	de	jaundice (potions)	
*Flemingia semialata* Roxb. [Chor ae go boe]	0.30	1187	S	F	Ma	L	no	wood coloring	15
					Me	R/W	bo/de	fatigue/jaundice (bath/potions)	85
*Flemingia stricta* Roxb. [Ae go per]	0.20	0987	S	F	Me	L	po	lactation stimulant (liniment)	100
						R	de	kidney/urethral stone (potions)	
**Flemingia strobilifera (L.) W.T.Aiton [Choe gol boe/Se jor bi]	0.22	1041	S	F	Me	W	bo/de	jaundice (bath/potions)	85
					NP	Fl/L	no	insect repellants	15
*Glycine*									
***Glycine max (L.) Merr. [Tor nor klee]	1.27	0625	H	A	F	Se	bo	snack/dessert	71
					FA	Se	sm	seasoning	28
					Me	Se	bo	diabetes (oral)	1
*Grona*									
*Grona heterocarpon* (L.) H.Ohashi & K.Ohashi	0.59	0686	S	F/V	Me	W	de	back pain/lumbago (potions)	98
[Sae ngee po]					SU	L	bo	herbal tea	2
*Huangtcia*									
*Huangtcia renifolia* (L.) H.Ohashi & K.Ohashi [Hya pa la sa bee]	0.53	0898	S	F/V	Me	R/W	de	urethral stones (potions)	100
						W	de	back pain/lumbago (potions)	
*Hultholia*									
*Hultholia mimosoides* (Lam.) E. Gagnon & G. P. Lewis [Ta ner sor do]	0.23	1169	C	A/F	F	Fl/L	no	vegetable	100
*Indigofera*									
*Indigofera caloneura* Kruz [Jui tuu]	0.03	1171	S	F	Me	L	po	itching (poultices)	100
						R	de	lumbago (potions)	
*Indigofera hendecaphylla Jacq. [Sae ngee po]	0.23	0655	H	F/V	Me	W	de	back pain/lumbago (potions)	100
						W	bo	puerperium (bath)	
***Indigofera tinctoria L. [Soe ya khwo]	0.47	0717	S	H/F	F	Fl/L	bo/no	vegetable	17
					Ma	L	sm	fabric coloring	40
					Me	L	po	fever (poultices)	43
*Lablab*									
***Lablab purpureus (L.) Sweet [Bo ba sa]	1.02	0762	C	A/H	F	Fr	bo/co	vegetable	94
					Me	R	no	toothache (chew)	6
*Leucaena*									
***Leucaena leucocephala (Lam.) de Wit [Po shui se]	1.16	0520	S	H	F	L/Se	no	vegetable	98
					Me	L	no	palpitation (oral)	2
*Millettia*									
*Millettia brandisiana* Kruz [Ye ji dor]	0.29	1168	T	F	F	Fl/L	bo/no	vegetable	92
					Me	B	no	aphthous ulcers (lozenge)	8
*Millettia caerulea* Baker [Pua wua dor]	0.50	0776	C	F	F	L	bo	vegetable	58
					Me	L	de	purgative/urethral stone (potions)	42
*Mimosa*									
**Mimosa diplotricha Sauvalle [Naa tor dae]	0.02	0642	H	A/V	Me	W	de	fever (potions)	100
						W	de	urethral stones (potions)	
*Mimosa pigra* L. [Nor wee mae pa doh]	0.04	0599	S	A/V	EU	W	no	fence	50
					F	L	co	vegetables	25
					Me	S	de	fever (potions)	25
*Mimosa pudica* L. [Nor wee mae]	0.40	0628	H	A/V	Me	R/W	de	urethral stones (potions)	100
						W	bo	fever (herbal stream)	
*Mucuna*									
*Mucuna macrocarpa* Wall. [Ri mue jue]	0.21	1001	C	F	F	Fl/L	bo	vegetable	10
					Ma	St	no	rope	58
					Me	St	sm	feet pain (poultices)	32
*Mucuna pruriens* (L.) DC. [Por lue sa]	0.03	1016	C	F	Me	R	de	asthma (potions)	100
*Pachyrhizus*									
***Pachyrhizus erosus (L.) Urb. [Nuaow cher]	0.96	0936	C	A	F	R	no	fruit	100
*Paraderris*									
*Paraderris elliptica* (Wallich) Adema [Glae hyu]	0.80	0918	C	F	F	Se	bo/co	vegetable	3
					Ma	St	no	rope	21
					Me	R/St	po	itching (liniment)	11
					NP	R	po	herbicide/insecticide	10
					VP	R	po	fish poisoning	55
*Parkia*									
**Parkia leiophylla Kruz [Se kwi mae]	0.04	0130	T	F	F	Se	no	vegetable	100
*Phanera*									
**Phanera ornata var. *kerrii* (Gagnep.) K.Larsen & S.S.Larsen [Poe na meu tu]	0.91	1189	C	F	Ma	St	no	rope	21
					Me	B/St	de	back pain/lumbago (potions)	50
					SU	L	mc	cigarette paper	29
*Phanera* sp. [Poe na mue tu]	0.66	0726	C	F	Ma	St	no	rope	25
					Me	B/St	de	tonic (potions)	58
					SU	L	mc	cigarette paper	17
*Phaseolus*									
***Phaseolus vulgaris L. [Po to sa]	0.83	1131	C	A	F	F/Se	bo/co	vegetable	100
				
*Phyllodium*									
**Phyllodium longipes (Craib) Schindl. [Yo hor mae]	0.51	0869	S	F	F	L	co	vegetable	13
					Me	L/R	de	jaundice/puerperium (potions)	87
*Phyllodium pulchellum* (L.) Desv. [Tii si yo hor mae]	0.34	0943	S	F	Me	R/W	de	urethral stones (potions)	68
					NP	W	no	insect repellant	32
*Phyllodium vestitum* Benth. [Tii si yo hor mae]	0.12	1188	S	F	Me	L	de	urethral stones (potions)	91
					NP	L	no	insect repellant	9
*Psophocarpus*									
***Psophocarpus tetragonolobus (L.) DC. [Bo ba per wi]	1.03	0858	C	H	F	F/Se	bo	vegetable	90
					Me	L	po	ulcers (poultices)	10
*Pterocarpus*									
*Pterocarpus macrocarpus* Kruz [Goe roe / Toe roe]	0.71	0567	T	F	F	L	co	vegetable	2
					Fu	St	cu	firewood	2
					Ma	St	cu	construction materials	71
					Me	B/E	no	toothache (chew)	20
					NP	B/St	mi	leech repellant for cattle	5
*Pueraria*									
*Pueraria candollei* var. *mirifica* (Airy Shaw & Suvat.) Niyomdham [Su ku pue]	0.09	1038	C	F	Me	R	de/ gr	bruises/wounds (potions/oral)	63
					SU	R	no	pray for rainfall or evict natural disaster	37
*Senegalia*									
*Senegalia catechu* (L.f.) P.J.H.Hurter & Mabb. [Se bo blae / Se jui]	0.67	1182	T	F	Me	E	no	coughs/make teeth strong (chew)	33
					SU	E	no	chew with betel nut	67
*Senegalia megaladena* (Desv.) Maslin, Seigler & Ebinger [Klae kwo]	0.53	0861	C	F	Me	L/St	po/sm	itching (liniment)	46
					NP	B/R	sm	insecticide	2
					VP	B/R	po	fish poisoning	52
*Senegalia pennata* (L.) Maslin [Po shui dor]	1.07	1058	C	H	F	L	co	vegetable	94
					Me	L	co	eyes tonic (eat)	1
					NP	L	po	insect repellant	3
					SU	St	no	evict the rain ritual	2
*Senegalia rugata* (Lam.) Britton & Rose [Phu che sa]	2.39	1055	S	F/V	F	F	no	fruit	15
					FA	L	no	sour taste seasoning	22
					Ma	Fr	ci	soap/shampoo	7
					Me	Fr	ci	food poisoning	12
					SU	Fr	dr	holy water for rituals	44
*Senna*									
***Senna alata (L.) Roxb. [Ya la moe]	1.16	0971	S	H	Me	L	bo/sm	itching (liniment)	100
						L/Fl	de	flatulence (potions)	
*Senna hirsuta* (L.) H.S.Irwin & Barneby [Toe si ka]	0.22	0934	S	V	F	Se	ro	snack	15
					Me	W	bo	fever/muscle relaxant (herbal stream)	75
					SU	L	sm	fermented tea leave	10
***Senna occidentalis (L.) Link [Peor na nor dor]	0.82	0923	S	V	F	L/Fr	bo	vegetable	82
					Me	W	bo/de	diarrhea (oral/potions)	18
*Senna siamea* (Lam.) H.S.Irwin & Barneby [Sa lae de]	0.83	0927	T	V	F	Fl/L	bo	vegetable	91
					Me	Fl/L	bo	hypertension (oral)	9
*Senna tora* (L.) Roxb. [Nor ji koe]	0.26	0924	S	V	F	L	bo	vegetable	48
					Ma	S	no	broom	4
					Me	Se	ro&ci	urethral stones (drink)	44
					F	Se	ro	herbal tea	4
*Tadehagi*									
*Tadehagi triquetrum* (L.) H.Ohashi [Sae jo bi]	0.74	0951	S	A/F	F	R	co	vegetable	5
					Me	W	de	back pain/lumbago (potions)	95
*Tamarindus*									
***Tamarindus indica L.	3.38	0568	T	H/V	F	Fr	no	fruit	30
[Sae mor glae]					FA	L	co	sour taste seasoning	27
					Fu	St	dr	fuels	4
					Ma	St	cu	cutting board	9
					Me	B/Fr	de/no	coughs (potions/oral)	17
					SU	Fr	mi	make cigarette	13
*Uraria*									
*Uraria oblonga* (Wall. ex Benth.) H.Ohashi & K.Ohashi [Sae hui boe]	0.64	0662	S	F/V	Me	B/R	po	aphthous ulcer (chew)	100
						R/W	bo/de	jaundice (bath/potion)	
						W	de	back pain/lumbago (potions)	
*Vigna*									
*Vigna dalzelliana (Kuntze) Verdc. [Se bae mee]	0.17	0651	C	F	F	Fr/L	bo/no	vegetable	87
					Me	Se	ro	tonic (oral)	13
**^,^***Vigna mungo (L.) Hepper [Poe toe su]	0.67	1174	C	A	F	Se	bo/co	vegetable	100
**^,^***Vigna radiata (L.) R.Wilczek [Se bae klee]	0.96	1189	C	A	F	Se	bo/co	vegetable	93
					FA	Se	sm	seasoning	5
					SU	L	no	put on pedestal for traditional ritual	2
**^,^***Vigna umbellata (Thunb.) Ohwi & H.Ohashi [Poe toe khwo]	0.71	0935	C	A/H	F	Fr/Se	bo/co	vegetable	100
***Vigna unguiculata (L.) Walp. [Poe toe sho]	0.94	0969	C	A	F	Fr/Se	co	vegetable	100
*Xylia*									
*Xylia xylocarpa* (Roxb.) Taub. [Pei]	0.83	0649	T	F	Ma	St	cu	construction materials	31
					Me	Fr	de	anemia (potions)	57
					F	Fr	bo	herbal tea	12

Life form: C = Climber; H = Herb; S = Shrub; T = Tree. Habitat: A = Agricultural area; F = Forest; H = Home-gardens; V = Village. Use Categories: AF = Animal food; EU = Environmental uses; F = Food; FA = Food additives; Fu = Fuels; Ma = Materials; Me = Medicines; NP = Non-vertebrate poisons; SU = Social uses; VP = Vertebrate poisons. Part Used: B = Bark; E = Exudates; Fl = Inflorescences; Fr = Fruit; L = Leaves; R = Roots; Se = Seeds; St = Stems; W = Whole plant. Method of preparation: bm = burned and milled; bo = boiled; bob = boiled with bamboo shoot; bu = burned; cu = cut; ci = cold infusion co = cooked; coc = cooked with chicken; cr = crushed; cw = crushed with water; de = decoction; dr = dried; fe = fermented; gr = grated with water; hl = herbal liquor; mc = make cigarette; ms = mix with salt; no = none; po = pounded; por = pounded with rice; pos = pounded with salt; pr = prickle; re = reamed; ro = roasted; sm = smoked; sq = squeezed; ts = take seed coat out. Species that are new records of traditional use are marked with *. Species that are new records of traditional use in Karen communities are marked with **. Species with global economic importance use are marked with ***.

## Supplementary Materials

The following are available online at https://www.mdpi.com/2223-7747/8/12/600/s1, Table S1: Calculation of Cultural Important Index (CI) values for legume species used in three Karen villages in northern Thailand, Table S2: Calculation of Fidelity Level (FL) values for 83 legume species used in three Karen villages in northern Thailand.
