# Supplementary material for: Traditional Uses of Leguminosae among the Karen in Thailand
Source: Plants (Basel). 2019 Dec 13;8(12):600. doi: 10.3390/plants8120600 (PMC6963713; doi:10.3390/plants8120600)
Supplement: Supplementary file 1 [file plants-08-00600-s001.pdf]

Supplementary Data

Table S1. Calculation of Cultural Important Index (CI) values for legume species used in three Karen villages in northern Thailand.

The Cultural Importance Index (CI) is calculated with the formula $CI = \sum(u = 1)^{NC} \sum(i=1)^{N} URui/N$ (NC is the total number of use categories for species i, URui is the total number of use reports of each category, and N is the total number of informants). UR(AF)=use reports for animal foods; UR(EU)=use reports for environmental uses; UR(F)=use reports for foods; UR(FA)=use reports for food additives; UR(Fu)=use reports for fuels; UR(Ma)=use reports for materials; UR(Me)=use reports for medicines; UR(NP)=use reports for non-vertebrate poisons; UR(SU)=use reports for social uses; UR(VP)=use reports for vertebrate poisons.

Species	UR(AF)	UR(EU)	UR(F)	UR(FA)	UR(Fu)	UR(Ma)	UR(Me)	UR(NP)	UR(SU)	UR(VP)	∑UR	N	CI
Tamarindus indica L.	0	0	139	35	12	26	53	0	39	0	304	90	3.38
Senegalia rugata (Lam.) Britton & Rose	0	0	51	29	0	15	26	0	94	0	215	90	2.39
Glycine max (L.) Merr.	0	0	81	32	0	0	1	0	0	0	114	90	1.27
Entada rheedii Spreng.	0	0	28	0	0	44	34	0	0	0	106	90	1.18
Senna alata (L.) Roxb.	0	0	0	0	0	2	104	0	0	0	106	90	1.18
Leucaena leucocephala (Lam.) de Wit	0	0	102	0	0	0	2	0	0	0	104	90	1.16
Senegalia pennata (L.) Maslin	0	0	90	0	0	0	1	3	2	0	96	90	1.07
Biancaea sappan (L.) Tod.	0	0	1	0	0	1	94	0	0	0	96	90	1.07
Psophocarpus tetragonolobus (L.) DC.	0	0	84	0	0	0	9	0	0	0	93	90	1.03
Lablab purpureus (L.) Sweet	0	0	86	0	0	0	6	0	0	0	92	90	1.02
Arachis hypogaea L.	0	0	90	0	0	0	0	0	0	0	90	90	1.00
Bauhinia variegata L.	0	0	89	0	0	0	0	0	0	0	89	90	0.99
Pachyrhizus erosus (L.) Urb.	0	0	86	0	0	0	0	0	0	0	86	90	0.96
Phaseolus aureus Roxb.	0	0	80	4	0	0	0	0	2	0	86	90	0.96
Vigna unguiculata (L.) Walp.	0	0	85	0	0	0	0	0	0	0	85	90	0.84
Adenanthera pavonina L.	0	0	64	0	0	9	9	0	0	0	82	90	0.91
Phanera ornata var. *kerrii* (Gagnep.) K.Larsen & S.S.Larsen.	0	0	0	0	0	17	41	0	24	0	82	90	0.91
Phaseolus vulgaris L.	0	0	75	0	0	0	0	0	0	0	75	90	0.83
Xylia xylocarpa (Roxb.) Taub.	0	0	7	0	0	23	43	0	2	0	75	90	0.83
Senna siamea (Lam.) H.S.Irwin & Barneby	0	0	68	0	0	0	7	0	0	0	75	90	0.83
Senna occidentalis (L.) Link	0	0	61	0	0	0	13	0	0	0	74	90	0.82
Afzelia xylocarpa (Kurz) Craib	0	0	5	0	0	6	54	0	7	0	72	90	0.80
Paraderris elliptica (Wallich) Adema	0	0	2	0	0	15	8	7	0	40	72	90	0.80
Cassia fistula L.	0	0	7	0	0	1	62	0	0	0	70	90	0.78
Albizia chinensis (Osbeck) Merr.	0	0	0	0	0	59	10	0	0	0	69	90	0.77

Table 1. ***Cont.***

Species	UR(AF)	UR(EU)	UR(F)	UR(FA)	UR(Fu)	UR(Ma)	UR(Me)	UR(NP)	UR(SU)	UR(VP)	∑UR	N	CI
Canavalia ensiformis (L.) DC.	0	0	68	0	0	0	0	0	0	0	68	90	0.76
Dalbergia cultrata Benth.	1	0	10	0	1	37	19	0	0	0	68	90	0.76
Tadehagi triquetrum (L.) H.Ohashi	0	0	3	0	0	0	64	0	0	0	67	90	0.74
Desmodium velutinum (Willd.) DC.	0	0	0	0	0	0	63	1	2	0	66	90	0.73
Vigna umbellata (Thunb.) Ohwi & H.Ohashi	0	0	64	0	0	0	0	0	0	0	64	90	0.71
Eriosema chinense Vogel	0	0	37	0	0	0	25	0	0	0	62	90	0.69
Pterocarpus macrocarpus Kruz	0	0	1	0	1	44	13	3	0	0	62	90	0.69
Cajanus cajan (L.) Millsp.	0	0	53	0	0	0	8	0	0	0	61	90	0.68
Senegalia catechu (L.f.) P.J.H.Hurter & Mabb.	0	0	0	0	0	0	20	0	40	0	60	90	0.67
Vigna mungo (L.) Hepper	0	0	60	0	0	0	0	0	0	0	60	90	0.67
Archidendron clypearia (Jack) I.C.Nielsen	0	0	0	0	2	13	41	3	0	0	59	90	0.66
Phanera sp.	0	0	0	0	0	15	34	0	10	0	59	90	0.66
Uraria oblonga (Wall. ex Benth.) H.Ohashi & K.Ohashi	0	0	0	0	0	0	58	0	0	0	58	90	0.64
Archidendron jiringa (Jack) I.C.Nielsen	0	0	51	0	0	0	6	0	0	0	57	90	0.63
Dalbergia ovata Benth	0	0	0	0	0	0	42	0	14	0	56	90	0.62
Grona heterocarpon (L.) H.Ohashi & K.Ohashi	0	0	1	0	0	0	52	0	0	0	53	90	0.59
Flemingia congesta Roxb. Ex W.T. Aiton	0	0	0	0	0	0	52	0	0	0	52	90	0.58
Aeschynomene americana L.	0	0	0	0	0	0	51	0	0	0	51	90	0.57
Senegalia megaladena (Desv.) Maslin, Seigler & Ebinger	0	0	0	0	0	0	22	1	0	25	48	90	0.53
Huangtcia renifolia (L.) H.Ohashi & K.Ohashi	0	0	0	0	0	0	48	0	0	0	48	90	0.53
Phyllodium longipes (Craib) Schindl.	0	0	6	0	0	0	40	0	0	0	46	90	0.51
Millettia caerulea Baker	0	0	26	0	0	0	19	0	0	0	45	90	0.50
Cajanus goensis Dalzell	0	0	44	0	0	0	0	0	0	0	44	90	0.42
Flemingia paniculata Benth.	0	0	0	0	0	0	43	0	0	0	43	90	0.49
Indigofera tinctoria L.	0	0	7	0	0	17	18	0	0	0	42	90	0.47
Erythrina subumbrans (Hassk.) Merr.	0	3	22	0	0	9	5	0	0	0	39	90	0.43
Mimosa pudica L.	0	0	0	0	0	0	36	0	0	0	36	90	0.40
Dunbaria bella Prain	0	0	30	0	0	0	4	0	0	0	34	90	0.38
Phyllodium pulchellum (L.) Desv.	0	0	0	0	0	0	21	10	0	0	31	90	0.34
Albizia procera (Roxb.) Benth.	0	0	14	0	0	5	10	0	0	0	29	90	0.32
Erythrina stricta Roxb.	0	0	14	0	0	13	1	0	0	0	28	90	0.31

Table 1. ***Cont.***

Species	UR(AF)	UR(EU)	UR(F)	UR(FA)	UR(Fu)	UR(Ma)	UR(Me)	UR(NP)	UR(SU)	UR(VP)	∑UR	N	CI
Flemingia semialata Roxb.	0	0	0	0	0	4	23	0	0	0	27	90	0.30
Millettia brandisiana Kruz	0	0	24	0	0	0	2	0	0	0	26	90	0.29
Senna tora (L.) Roxb.	0	0	11	0	0	1	10	0	1	0	23	90	0.26
Bauhinia purpurea L.	0	0	21	0	0	0	0	0	0	0	21	90	0.23
Hultholia mimosoides (Lam.) E. Gagnon & G. P. Lewis	0	0	21	0	0	0	0	0	0	0	21	90	0.23
Crotalaria bracteata DC.	0	0	0	0	0	0	18	0	3	0	21	90	0.23
Indigofera hendecaphylla Jacq.	0	0	0	0	0	0	21	0	0	0	21	90	0.23
Flemingia strobilifera (L.) W.T.Aiton	0	0	0	0	0	0	17	3	0	0	20	90	0.22
Senna hirsuta (L.) H.S.Irwin & Barneby	0	0	3	0	0	0	15	0	2	0	20	90	0.22
Mucuna macrocarpa Wall.	0	0	2	0	0	11	6	0	0	0	19	90	0.21
Flemingia stricta Roxb.	0	0	0	0	0	0	18	0	0	0	18	90	0.20
Crotalaria lejoloba Bartl.	0	0	1	0	0	0	13	0	3	0	17	90	0.19
Canavalia gladiata (Jacq.) DC.	0	0	16	0	0	0	0	0	0	0	16	90	0.18
Flemingia lineata (L.) Aiton	0	0	0	0	0	0	16	0	0	0	16	90	0.18
Crotalaria alata D.Don	0	0	1	0	0	2	10	0	3	0	16	90	0.18
Vigna dalzelliana (Kuntze) Verdc.	0	0	13	0	0	0	2	0	0	0	15	90	0.17
Dalbergia stipulacea Roxb.	0	0	0	0	1	7	6	0	0	0	14	90	0.16
Phyllodium vestitum L.	0	0	0	0	0	0	10	1	0	0	11	90	0.12
Crotalaria pallida Aiton	0	0	1	0	0	0	8	0	1	0	10	90	0.11
Pueraria candollei var. *mirifica* (Airy Shaw & Suvat.) Niyomdham	0	0	0	0	0	0	5	0	3	0	8	90	0.09
Crotalaria albida Roth	0	0	0	0	0	0	4	0	0	0	4	90	0.04
Crotalaria sessiliflora L.	0	0	0	0	0	0	4	0	0	0	4	90	0.04
Mimosa pigra L.	0	0	1	0	0	2	1	0	0	0	4	90	0.04
Parkia leiophylla Kruz	0	0	4	0	0	0	0	0	0	0	4	90	0.04
Indigofera caloneura Kurz	0	0	0	0	0	0	3	0	0	0	3	90	0.03
Mucuna pruriens (L.) DC.	0	0	0	0	0	0	3	0	0	0	3	90	0.03
Mimosa diplotricha Sauvalle	0	0	0	0	0	0	2	0	0	0	2	90	0.02

Table 2. Calculation of Fidelity Level (FL) values for 83 legume species used in three Karen villages in northern Thailand.

FL is calculated as: FL (%) = (Np/N) x 100 where Np is the number of use-reports in each of the use categories and N is the total number of use-report. The Use Categories were: AF = animal foods; EU = environmental uses; F = foods; FA = food additives; Fu = fuels; Ma = materials; Me = medicines; NP = non-vertebrate poisons; SU = social uses; VP = vertebrate poisons.

**Species**	**AF**		**EU**		**F**		**FA**		**Fu**		**Ma**		**Me**		**NP**		**SU**		**VP**		**N**
	**Np**	**FL**	**Np**	**FL**	**Np**	**FL**	**Np**	**FL**	**Np**	**FL**	**Np**	**FL**	**Np**	**FL**	**Np**	**FL**	**Np**	**FL**	**Np**	**FL**	
***Adenanthera pavonina* L.**	0	0	0	0	64	78	0	0	0	0	9	11	9	11	0	0	0	0	0	0	82
Aeschynomene americana L.	0	0	0	0	0	0	0	0	0	0	0	0	51	100	0	0	0	0	0	0	51
Afzelia xylocarpa (Kurz) Craib	0	0	0	0	5	7	0	0	0	0	6	8	54	75	0	0	7	10	0	0	72
Albizia chinensis (Osbeck) Merr.	0	0	0	0	0	0	0	0	0	0	59	86	10	14	0	0	0	0	0	0	69
Albizia procera (Roxb.) Benth.	0	0	0	0	14	48	0	0	1	3	4	14	10	34	0	0	0	0	0	0	29
Arachis hypogaea L.	0	0	0	0	90	100	0	0	0	0	0	0	0	0	0	0	0	0	0	0	90
Archidendron clypearia (Jack) I.C.Nielsen	0	0	0	0	0	0	0	0	2	3	13	22	41	69	3	5	0	0	0	0	59
Archidendron jiringa (Jack) I.C.Nielsen	0	0	0	0	51	89	0	0	0	0	0	0	6	11	0	0	0	0	0	0	57
Bauhinia purpurea L.	0	0	0	0	21	100	0	0	0	0	0	0	0	0	0	0	0	0	0	0	21
Bauhinia variegata L.	0	0	0	0	89	100	0	0	0	0	0	0	0	0	0	0	0	0	0	0	89
Biancaea sappan (L.) Tod.	0	0	0	0	1	1	0	0	0	0	1	1	94	98	0	0	0	0	0	0	96
Cajanus cajan (L.) Millsp.	0	0	0	0	53	87	0	0	0	0	0	0	8	13	0	0	0	0	0	0	61
Cajanus goensis Dalzell	0	0	0	0	44	100	0	0	0	0	0	0	0	0	0	0	0	0	0	0	44
Canavalia ensiformis (L.) DC.	0	0	0	0	68	100	0	0	0	0	0	0	0	0	0	0	0	0	0	0	68
Canavalia gladiata (Jacq.) DC.	0	0	0	0	16	100	0	0	0	0	0	0	0	0	0	0	0	0	0	0	16
Cassia fistula L.	0	0	0	0	7	10	0	0	0	0	1	1	62	89	0	0	0	0	0	0	70
Crotalaria alata D.Don	0	0	0	0	1	6	0	0	0	0	2	13	10	63	0	0	3	19	0	0	16
Crotalaria albida Roth	0	0	0	0	0	0	0	0	0	0	0	0	4	100	0	0	0	0	0	0	4
Crotalaria bracteata DC.	0	0	0	0	0	0	0	0	0	0	0	0	18	86	0	0	3	14	0	0	21
Crotalaria lejoloba Bartl.	0	0	0	0	1	6	0	0	0	0	0	0	13	76	0	0	3	18	0	0	17
Crotalaria pallida Aiton	0	0	0	0	1	10	0	0	0	0	0	0	8	80	0	0	1	10	0	0	10
Crotalaria sessiliflora L.	0	0	0	0	0	0	0	0	0	0	0	0	4	100	0	0	0	0	0	0	4
Dalbergia cultrata Benth.	1	1	0	0	10	15	0	0	1	1	37	54	19	28	0	0	0	0	0	0	68
Dalbergia ovata Benth.	0	0	0	0	0	0	0	0	0	0	0	0	42	75	0	0	14	25	0	0	56
Dalbergia stipulacea Roxb.	0	0	0	0	0	0	0	0	1	7	7	50	6	43	0	0	0	0	0	0	14
Desmodium velutinum (Willd.) DC.	0	0	0	0	0	0	0	0	0	0	0	0	63	98	1	2	0	0	0	0	64

Table 2. *Cont.*

Species	AF		EU		F		FA		Fu		Ma		Me		NP		SU		VP		N
	Np	FL	Np	FL	Np	FL	Np	FL	Np	FL	Np	FL	Np	FL	Np	FL	Np	FL	Np	FL	
Dunbaria bella Prain	0	0	0	0	30	88	0	0	0	0	0	0	4	12	0	0	0	0	0	0	34
Entada rheedii Spreng.	0	0	0	0	28	26	0	0	0	0	44	41	34	31	0	0	2	2	0	0	108
Eriosema chinense Vogel	0	0	0	0	35	56	0	0	0	0	0	0	25	40	0	0	2	3	0	0	62
Erythrina stricta Roxb.	0	0	0	0	14	50	0	0	0	0	13	46	1	4	0	0	0	0	0	0	28
Erythrina subumbrans (Hassk.) Merr.	0	0	3	8	22	56	0	0	0	0	9	23	5	13	0	0	0	0	0	0	39
Flemingia congesta Roxb. Ex W.T. Aiton	0	0	0	0	0	0	0	0	0	0	0	0	52	100	0	0	0	0	0	0	52
Flemingia lineata (L.) Aiton	0	0	0	0	0	0	0	0	0	0	0	0	16	100	0	0	0	0	0	0	16
Flemingia semialata Roxb.	0	0	0	0	0	0	0	0	0	0	4	15	23	85	0	0	0	0	0	0	27
Flemingia stricta Roxb.	0	0	0	0	0	0	0	0	0	0	0	0	18	100	0	0	0	0	0	0	18
Flemingia strobilifera (L.) W.T.Aiton	0	0	0	0	0	0	0	0	0	0	0	0	17	85	3	15	0	0	0	0	20
Flemingia paniculata Benth.	0	0	0	0	0	0	0	0	0	0	0	0	43	100	0	0	0	0	0	0	43
Glycine max (L.) Merr.	0	0	0	0	81	71	32	28	0	0	0	0	1	1	0	0	0	0	0	0	114
Grona heterocarpon (L.) H.Ohashi & K.Ohashi	0	0	0	0	0	0	0	0	0	0	0	0	52	98	0	0	1	2	0	0	53
Huangtcia renifolia (L.) H.Ohashi & K.Ohashi	0	0	0	0	0	0	0	0	0	0	0	0	48	100	0	0	0	0	0	0	48
Hultholia mimosoides (Lam.) E. Gagnon & G. P. Lewis	0	0	0	0	21	100	0	0	0	0	0	0	0	0	0	0	0	0	0	0	21
Indigofera caloneura Kruz.	0	0	0	0	0	0	0	0	0	0	0	0	3	100	0	0	0	0	0	0	3
Indigofera hendecaphylla Jacq.	0	0	0	0	0	0	0	0	0	0	0	0	21	100	0	0	0	0	0	0	21
Indigofera tinctoria L.	0	0	0	0	7	17	0	0	0	0	17	40	18	43	0	0	0	0	0	0	42
Lablab purpureus (L.) Sweet	0	0	0	0	86	93	0	0	0	0	0	0	6	7	0	0	0	0	0	0	92
Leucaena leucocephala (Lam.) de Wit	0	0	0	0	102	98	0	0	0	0	0	0	2	2	0	0	0	0	0	0	104
Millettia brandisiana Kruz	0	0	0	0	24	92	0	0	0	0	0	0	2	8	0	0	0	0	0	0	26
Millettia caerulea Baker	0	0	0	0	26	58	0	0	0	0	0	0	19	42	0	0	0	0	0	0	45
Mimosa diplotricha Sauvalle	0	0	0	0	0	0	0	0	0	0	0	0	2	100	0	0	0	0	0	0	2
Mimosa pigra L.	0	0	2	50	1	25	0	0	0	0	0	0	1	25	0	0	0	0	0	0	4
Mimosa pudica L.	0	0	0	0	0	0	0	0	0	0	0	0	36	100	0	0	0	0	0	0	36
Mucuna macrocarpa Wall.	0	0	0	0	2	11	0	0	0	0	11	58	6	32	0	0	0	0	0	0	19
Mucuna pruriens (L.) DC.	0	0	0	0	0	0	0	0	0	0	0	0	3	100	0	0	0	0	0	0	3

Table 2. *Cont.*

Species	AF		EU		F		FA		Fu		Ma		Me		NP		SU		VP		N
	Np	FL	Np	FL	Np	FL	Np	FL	Np	FL	Np	FL	Np	FL	Np	FL	Np	FL	Np	FL	
Pachyrhizus erosus (L.) Urb.	0	0	0	0	86	100	0	0	0	0	0	0	0	0	0	0	0	0	0	0	86
Paraderris elliptica (Wallich) Adema	0	0	0	0	2	3	0	0	0	0	15	21	8	11	7	10	0	0	40	56	72
Parkia leiophylla Kruz	0	0	0	0	4	100	0	0	0	0	0	0	0	0	0	0	0	0	0	0	4
Phanera ornata var. *kerrii* (Gagnep.) K.Larsen & S.S.Larsen	0	0	0	0	0	0	0	0	0	0	17	21	41	50	0	0	24	29	0	0	82
Phanera sp.	0	0	0	0	0	0	0	0	0	0	15	25	34	58	0	0	10	17	0	0	59
Phaseolus vulgaris L.	0	0	0	0	75	100	0	0	0	0	0	0	0	0	0	0	0	0	0	0	75
Phyllodium longipes (Craib) Schindl.	0	0	0	0	6	13	0	0	0	0	0	0	40	87	0	0	0	0	0	0	46
Phyllodium pulchellum (L.) Desv.	0	0	0	0	0	0	0	0	0	0	0	0	21	68	10	32	0	0	0	0	31
Phyllodium vestitum Benth.	0	0	0	0	0	0	0	0	0	0	0	0	10	91	1	9	0	0	0	0	11
Psophocarpus tetragonolobus (L.) DC.	0	0	0	0	84	90	0	0	0	0	0	0	9	10	0	0	0	0	0	0	93
Pterocarpus macrocarpus Kruz	0	0	0	0	1	2	0	0	1	2	46	72	13	20	3	5	0	0	0	0	64
Pueraria candollei var. *mirifica* (Airy Shaw & Suvat.) Niyomdham	0	0	0	0	0	0	0	0	0	0	0	0	5	63	0	0	3	38	0	0	8
Senegalia catechu (L.f.) P.J.H.Hurter & Mabb.	0	0	0	0	0	0	0	0	0	0	0	0	20	33	0	0	40	67	0	0	60
Senegalia megaladena (Desv.) Maslin, Seigler & Ebinger	0	0	0	0	0	0	0	0	0	0	0	0	22	46	1	2	0	0	25	52	48
Senegalia pennata (L.) Maslin	0	0	0	0	90	94	0	0	0	0	0	0	1	1	3	3	2	2	0	0	96
Senegalia rugata (Lam.) Britton & Rose	0	0	0	0	32	15	48	22	0	0	15	7	26	12	0	0	94	44	0	0	215
Senna alata (L.) Roxb.	0	0	0	0	0	0	0	0	0	0	0	0	104	100	0	0	0	0	0	0	104
Senna hirsuta (L.) H.S.Irwin & Barneby	0	0	0	0	3	15	0	0	0	0	0	0	15	75	0	0	2	10	0	0	20
Senna occidentalis (L.) Link	0	0	0	0	61	82	0	0	0	0	0	0	13	18	0	0	0	0	0	0	74
Senna siamea (Lam.) H.S.Irwin & Barneby	0	0	0	0	68	91	0	0	0	0	0	0	7	9	0	0	0	0	0	0	75
Senna tora (L.) Roxb.	0	0	0	0	11	48	0	0	0	0	1	4	10	43	0	0	1	4	0	0	23

Table 2. *Cont.*

Species	AF		EU		F		FA		Fu		Ma		Me		NP		SU		VP)		N
	Np	FL	Np	FL	Np	FL	Np	FL	Np	FL	Np	FL	Np	FL	Np	FL	Np	FL	Np	FL	
Tadehagi triquetrum (L.) H.Ohashi	0	0	0	0	3	4	0	0	0	0	0	0	64	96	0	0	0	0	0	0	67
Tamarindus indica L.	0	0	0	0	90	30	84	28	12	4	26	9	53	17	0	0	39	13	0	0	304
Uraria oblonga (Wall. ex Benth.) H.Ohashi & K.Ohashi	0	0	0	0	0	0	0	0	0	0	0	0	58	100	0	0	0	0	0	0	58
Vigna dalzelliana (Kuntze) Verdc	0	0	0	0	13	87	0	0	0	0	0	0	2	13	0	0	0	0	0	0	15
Vigna mungo (L.) Hepper	0	0	0	0	60	100	0	0	0	0	0	0	0	0	0	0	0	0	0	0	60
Vigna radiata (L.) R.Wilczek	0	0	0	0	80	93	4	5	0	0	0	0	0	0	0	0	2	2	0	0	86
Vigna umbellata (Thunb.) Ohwi & H.Ohashi	0	0	0	0	64	100	0	0	0	0	0	0	0	0	0	0	0	0	0	0	64
Vigna unguiculata (L.) Walp.	0	0	0	0	85	100	0	0	0	0	0	0	0	0	0	0	0	0	0	0	85
Xylia xylocarpa (Roxb.) Taub.	0	0	0	0	0	0	0	0	0	0	23	31	43	57	0	0	9	12	0	0	75
